# Association of rare and common genetic variants in *MOCOS* with inadequate response to allopurinol

**DOI:** 10.1093/rheumatology/keae420

**Published:** 2024-08-13

**Authors:** Niamh C Fanning, Murray Cadzow, Ruth K Topless, Chris Frampton, Nicola Dalbeth, Tony R Merriman, Lisa K Stamp

**Affiliations:** Department of Medicine, University of Otago, Christchurch, Aotearoa, New Zealand; Department of Biochemistry, University of Otago, Dunedin, Aotearoa, New Zealand; Research and Teaching IT Support, University of Otago, Dunedin, Aotearoa, New Zealand; Department of Biochemistry, University of Otago, Dunedin, Aotearoa, New Zealand; Department of Medicine, University of Otago, Christchurch, Aotearoa, New Zealand; Department of Medicine, University of Auckland, Auckland, Aotearoa, New Zealand; Department of Biochemistry, University of Otago, Dunedin, Aotearoa, New Zealand; Division of Clinical Immunology and Rheumatology, University of Alabama at Birmingham, Birmingham, AL, USA; Department of Medicine, University of Otago, Christchurch, Aotearoa, New Zealand

**Keywords:** gout, allopurinol, drug response, genetics

## Abstract

**Objectives:**

The minor allele of the common rs2231142 *ABCG2* variant predicts inadequate response to allopurinol urate lowering therapy. We hypothesize that additional variants in genes encoding urate transporters and allopurinol-to-oxypurinol metabolic enzymes also predict allopurinol response.

**Methods:**

This study included a subset of participants with gout from the Long-term Allopurinol Safety Study Evaluating Outcomes in Gout Patients (LASSO), whose whole genome was sequenced (*n* = 563). Good responders had a 4:1 or 5:1 ratio of good [serum urate (SU) <0.36 mmol/l on allopurinol ≤300 mg/day] to poor (SU ≥0.36 mmol/l despite allopurinol >300 mg/day) responses over five to six time points, while inadequate responders had a 1:4 or 1:5 ratio of good to poor responses. Adherence to allopurinol was determined by pill counts, and for a subgroup (*n* = 303), by plasma oxypurinol >20μmol/l. Using the sequence kernel association test (SKAT), we estimated the combined effect of rare and common variants in urate secretory (*ABCC4*, *ABCC5*, *ABCG2*, *SLC17A1*, *SLC17A3*, *SLC22A6*, *SLC22A8*) and reuptake genes (*SLC2A9*, *SLC22A11*) and in allopurinol-to-oxypurinol metabolic genes (*AOX1*, *MOCOS*, *XDH*) on allopurinol response.

**Results:**

There was an association of rare and common variants in the allopurinol-to-oxypurinol gene group (*P*_SKAT-C_ = 0.019), and in *MOCOS*, encoding molybdenum cofactor sulfurase, with allopurinol response (*P*_SKAT-C_ = 0.011). Evidence for genetic association with allopurinol response in the allopurinol-to-oxypurinol gene group (*P*_SKAT-C_ = 0.002) and *MOCOS* (*P*_SKAT-C_ < 0.001) was stronger when adherence to allopurinol therapy was confirmed by plasma oxypurinol.

**Conclusion:**

We provide evidence for common and rare genetic variation in *MOCOS* associating with allopurinol response.

Rheumatology key messages

- Common and rare genetic variation in allopurinol metabolic gene, *MOCOS*, predicts allopurinol response in gout
- Higher serum urate and number of gout flares before allopurinol therapy were associated with inadequate response

## Introduction

**Figure 1.**
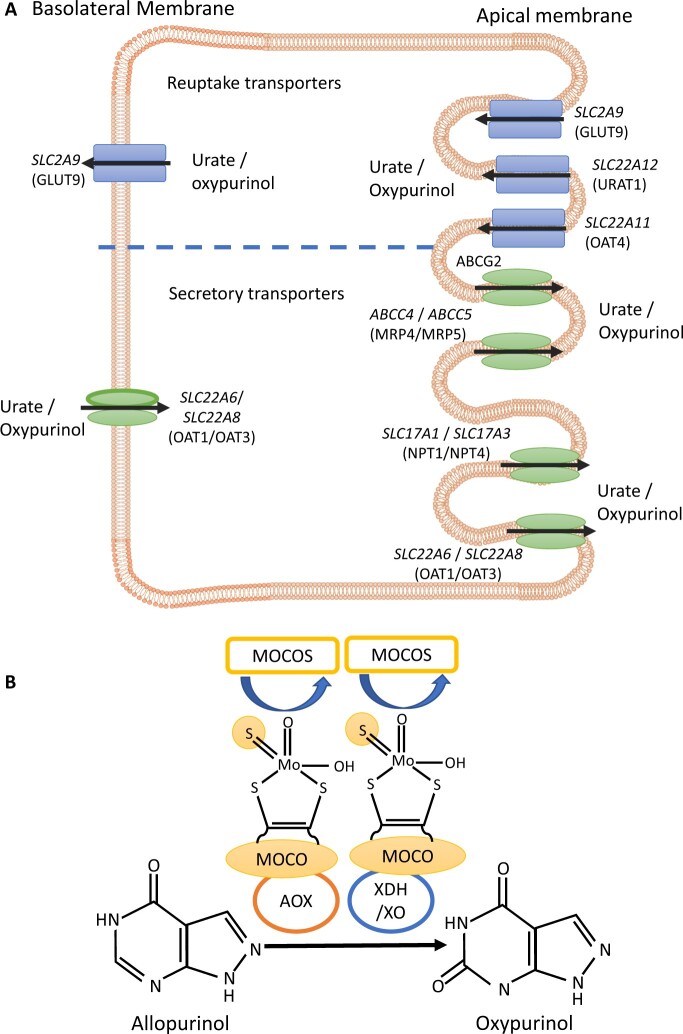
Genes encoding urate transporter proteins and enzymes involved in allopurinol metabolism may influence response to allopurinol therapy. (**A**) Urate reuptake proteins [shown in blue; URAT1 (gene: *SLC22A12*), GLUT9 (*SLC2A9*), OAT4 (*SLC22A11*)] and secretory transporter proteins [shown in green: MRP4 (*ABCC4*), MRP5 (*ABCC5*), ABCG2 (*ABCG2*), NPT1 (*SLC17A1*), NPT4 (*SLC17A3*), OAT1 (*SLC22A6*), OAT3 (*SLC22A8*)] in proximal tubule epithelial cells of the kidney. Urate transporter proteins are involved in reabsorption and secretion of urate and oxypurinol, the active metabolite of allopurinol. (**B**) Metabolism of allopurinol. Xanthine oxidase (XO) and aldehyde oxidase (AOX) metabolize the conversion of allopurinol to oxypurinol. XO and AOX activity is dependent on sulfuration of molybdenum cofactor (MOCO). Sulfuration of MOCO is mediated by MOCO sulfurase (MOCOS)

Hyperuricaemia, the precursor to gout, is primarily caused by under-excretion of urate by the kidneys and gut [1]. Urate excretion and reabsorption is regulated by secretory and reuptake transporters (Fig. 1A) [1]. Examples of reuptake transporters include GLUT9 and OAT4, encoded by *SLC2A9* and *SLC22A11*, respectively, which are essential for trans-epithelial urate reabsorption. Proteins implicated in urate secretion include ATP-driven efflux pump proteins, ABCG2 and MRP4 (*ABCC4*), which secrete urate via the proximal tubule and intestine. Additionally, sodium-dependent phosphate transporter proteins, NPT1 (*SLC17A1*) and NPT4 (*SLC17A3*), mediate electrogenic urate secretion via the renal apical membrane [1]. Age [2], sex [2, 3], body mass index (BMI) [4], gout flares [5] and ancestry [6] influence the effects of genetic variants in urate transporter genes.

Reducing and maintaining serum urate (SU) levels below 0.36 mmol/l is key to gout management [1]. This usually requires urate-lowering therapy (ULT), with allopurinol recommended as the first-line ULT. After absorption through the gut, allopurinol is rapidly converted by aldehyde oxidase (enzyme classification, EC 1.2.3.1; AOX) to oxypurinol [7]. Oxypurinol inhibits xanthine oxidoreductase (EC 1.1.1.204 and EC 1.1.3.22; XOR) a key enzyme in purine catabolism. XOR exists in two forms, xanthine oxidase (EC 1.17.3.2; XO) and xanthine dehydrogenase (EC 1.17.1.4; XDH), both are forms of the same gene product encoded by *XDH* [8]. XO/XDH converts allopurinol to oxypurinol at a lower rate than AOX (Fig. 1B) [7]. XO and AOX activity is dependent on sulfuration of molybdenum cofactor (MOCO) by MOCO sulfurase (MOCOS) (Fig. 1B) [7, 9]. Evidence suggests that oxypurinol excretion and reabsorbption via the kidneys is mediated by urate transporters [7].

Many people with gout never reach target SU on allopurinol [7], leading to risk of recurrent gout flares [10]. For most this is due to non-adherence to treatment or under-dosing. However, in some people target SU levels are not reached despite high doses of allopurinol and adherence confirmed by plasma oxypurinol concentrations [7]. Current understanding of why allopurinol treatment efficacy varies among individuals with gout is limited. Plasma oxypurinol levels vary between individuals receiving the same dose of allopurinol, suggesting differences in metabolism and or excretion of allopurinol and oxypurinol [11]. There is evidence that BMI, creatinine clearance and baseline SU associate with allopurinol response and metabolism [12], and evidence that age, sex, weight, estimated glomerular filtration rate (eGFR) and diuretic use associate with plasma oxypurinol levels [13]. Post-hoc analysis of the STOP Gout trial provided evidence that several patient-level characteristics (older age, higher education, quality of life measures) predict achievement of SU below target on ULT, as well as patient-level characteristics (non-White race, higher baseline SU, tophi presence and diuretic use) that predict failure to reach SU below target [14]. Genome-wide association studies (GWAS) identified the rs2231142 genetic variant in *ABCG2* as a predictor of inadequate response to allopurinol [15]. This was replicated in a cohort with plasma oxypurinol-confirmed adherence to allopurinol [16]. While GWAS are limited to predicting associations of common variants, genome and exome sequencing data can be used to identify cumulative effects of common and rare variants [17]. The goal of this study was to test an a priori hypothesis that common and rare variation in genes with roles in allopurinol metabolism and secretion and reuptake of urate and/or oxypurinol are associated with response to allopurinol.

## Methods

### Participant recruitment

This study included cross sectional data of a subset of participants (*n* = 667 of the total 1735) enrolled in the Long-term Allopurinol Safety Study Evaluating Outcomes in Gout Patients (LASSO) (NCT01391325), whose whole genomes were sequenced [18]. LASSO was a large, multicentre study to evaluate safety of allopurinol dose titration. Between June 2011 and January 2013, the LASSO trial enrolled adults aged 18–85 fulfilling the American Rheumatism Association preliminary classification criteria for gout and with ≥2 flares in the past year [19]. LASSO exclusion criteria included baseline SU <0.387 mmol/l (exclusion/inclusion criteria are detailed in Supplementary Table S1, available at *Rheumatology* online). The current study included 667 LASSO participants with peripheral blood samples available for whole genome sequencing (WGS) and who consented to genetic analysis. Supplementary Table S2, available at *Rheumatology* online, shows participant characteristics of the WGS 667 subset compared with other LASSO participants. Participant exclusion steps (detailed below) left 563 of 667 participants for analysis (Supplementary Fig. S1B, available at *Rheumatology* online). In the original study, investigators were encouraged but not required to increase allopurinol dose to achieve SU <0.36 mmol/l. Allopurinol dose and SU were collected over five or six monthly time points. Each participant visit was classified as a good (SU <0.36 mmol/l on allopurinol ≤300 mg/day) or poor (SU ≥0.36 mmol/l despite allopurinol >300 mg/day) response. Participants were classified into response groups based on the ratio of good to poor response visits. Good responders had ≥1 good and no poor response visits; or a 4:1 or 5:1 good: poor response visit ratio. Inadequate responders had ≥1 poor and no good response visits; or a 1:4 or 1:5 good: poor response visit ratio. Adherence to allopurinol was determined by pill counting and only visits with >80% compliance were used in response classification. Plasma samples were obtained at a single visit for a subset of 303 participants for whom plasma oxypurinol was measured.

All participants provided written informed consent, and ethical approval was obtained from the United States Schulman Central Institutional Review Board (201102890).

### Sample ascertainment and sequencing

WGS was performed from peripheral blood-derived DNA at the Garvan Institute (Sydney) using Illumina technology and at ∼30X coverage.

### Variant calling, annotation and filtering

Sequencing reads were aligned to the Hg19/b37 reference genome with the Burrows–Wheeler alignment tool [20], and were analysed with the Genome Analysis Toolkit (GATK) v3.6 for variant quality score recalibration (VQSR), indel realignment and duplicate removal. Single nucleotide polymorphism (SNP) and indel discovery and genotyping were performed simultaneously across all samples using VQSR according to GATK best practices recommendations [21, 22]. Variant call format (VCF) files were processed using Bcftools v1.10.2 [23]. VCFs were filtered to contain exonic variants within the TruSeq DNA Exome Illumina target region (615 808 variants). Variant-level filtering steps are shown in Supplementary Fig. S1A, available at *Rheumatology* online. The GATK VQSR PASS filter was applied to include variants that met the GATK-recommended truth sensitivity threshold. SNPs within 10 bp of indels were excluded. Genotypes with read depth <10 or genotype quality <20 were marked missing. Variants with Hardy–Weinberg equilibrium (HWE) *p* < 1 × 10^−6^ or >10% missing genotypes were excluded. The VCF file was normalized, multiallelic calls split into biallelic variants and indels left-aligned. Variant rsIDs were added using SNPSift and dbSNP build 155 with Hg19/b37 reference genome coordinates [24]. Predicted effect annotations were added using SnpEff and Hg19/b37 coordinates [25]. Functional prediction annotations from dbNSFP version 4.2a (using Hg19 coordinates) were added using SnpSift [24]. Pfam (protein families) domain annotations were searched using VarSite [26, 27]. The VCF was filtered to contain variants within 13 genes of interest (587 variants) (Supplementary Table S3, available at *Rheumatology* online). Genes were grouped by function: allopurinol-to-oxypurinol metabolic genes (*AOX1*, *MOCOS*, *XDH*); reuptake transporter genes (*SLC22A12*, *SLC2A9*, *SLC22A11*) and secretory transporter genes (*ABCC4*, *ABCC5*, *ABCG2*, *SLC17A1*, *SLC17A3*, *SLC22A6*, *SLC22A8*). The established allopurinol response *ABCG2* variant rs2231142 (15, 16) was analysed separately. Variants with minor allele frequency (MAF) ≥0.05 were pruned for linkage disequilibrium (LD) based on within-cohort metrics using PLINK version 1.90b6.13, an *r^2^* cut-off of 0.5, a window size of 50 variants and a sliding window size of five variants (23 of 587 variants were excluded) [28]. Only variants with HIGH or MODERATE impact according to the SnpEFF Putative_impact sub-field were included in analyses (225 of 564 variants, Supplementary Table S4, available at *Rheumatology* online) [25]. Six singleton variants unreported in dbSNP build 155 were excluded (Supplementary Fig. S1A, available at *Rheumatology* online). This left 219 variants within 12 genes (there were no variants in *SLC22A12*), the majority (193 of 219) being missense variants (Supplementary Table S4, available at *Rheumatology* online). The alternate allele in the reference genome was assumed to be the minor allele, unless the alternate allele frequency was >0.5 whereupon it was assumed to be the major allele. Variant genotypes were coded according to minor allele count (0: homozygous for major allele; 1: heterozygous; or 2: homozygous for minor allele).

### Individual-level filtering

Individual-level filters were based on exome data (Supplementary Fig. S1B, available at *Rheumatology* online). Participant samples with >10% missing genotypes were excluded (*n* = 25). Individuals with a percentage of heterozygous concordance <98% with array genotyping results (obtained using the Illumina Infinium CoreExome v24 bead chip platform [29]) were excluded (*n* = 65). Identical by descent (IBD) and principal component (PC) analyses were conducted using PLINK v1.9 [28], following LD pruning of common variants (MAF >0.5) using a 50 kb window size, five variant sliding window and an *r*^2^ = 0.1 threshold. Pairwise IBD coefficients were calculated from the pruned set of 15 647 markers. Six outlier pairs on Z0 *vs* Z1 coefficient plots were identified, which had PI_HAT >0.375 indicating duplicate samples or first-degree relatedness. The sample with lower heterozygous genotype concordance from each pair was excluded (*n* = 6). Exome-wide PC eigenvectors were calculated using pruned data (15 687 markers).

Eight participants missing age, BMI or eGFR data were excluded. Mean [standard deviation (s.d.)] singleton genotype concordance across included samples (*n* = 563) was 99.47 (1.33).

Mismatches between self-reported and PCA-defined ancestry were excluded previously, by clustering the first four genome-wide PC eigenvectors calculated previously with 2858 markers, genotyped on the Illumina Infinium CoreExome v24 bead chip platform (Illumina, Inc., San Diego, CA, USA) [29].

### Statistical analysis

Statistical analyses were performed using R statistical software version 4.0.2. (R Core Team 2018) [30]. Pearson's chi-square test and Wilcoxon’s rank sum test were used to calculate *P*-values of association with allopurinol response for categorical and continuous variables, respectively. The sequence kernel association test (SKAT) is a regression method that uses a supervised machine-learning to estimate associations between genetic variants in a region with a trait [17]. The SKAT R package was used to test the combined effect of common and rare variants within each gene-type group or gene on inadequate response to allopurinol [17]. Firstly, the SKAT_Null_Model function was used to compute model parameters and residuals for SKAT with allopurinol response as the dependent variable. Covariables incorporated in the SKAT_Null_Model for statistical adjustment— age, sex, BMI, eGFR, number of gout flares (past year), diuretic use, *ABCG2* rs2231142 and the top 5 exome-wide principal components (to control for population structure and ancestry)—were selected based on evidence from the literature of association with allopurinol response (dependent variable), and allopurinol metabolism/plasma oxypurinol level (independent variable) or uric acid transporters (independent variable). Small sample adjustment was applied using random sampling to estimate the kurtosis of the test statistics. Secondly, the SKAT_CommonRare (SKAT-C) function with default settings was applied to sequencing genotype data using the output object of the SKAT_Null_Model. Default weights were β(1,25) and β(0.5,0.5) for rare and common variants, respectively. The default common-rare cut off was $\frac{1}{\sqrt{2\times \mathrm{sample}\;\mathrm{size}}}$. Markers with >0.15 missingness or minor allele count = 0 were excluded from SKAT-CommonRare (SKAT-C) tests. Missing genotypes were imputed based on HWE by assigning the mean genotype value (2*p*, *p* is the MAF). A statistical significance threshold of *P* < 0.05 was applied to results of a priori SKAT-C tests of gene-type groups (secretory, reuptake and allopurinol-to-oxypurinol) for association with allopurinol response. Where significant SKAT-C associations were observed for a gene-type, posteriori SKAT-C tests were carried out for individual genes within that group, and a Bonferroni-corrected significance threshold based on the number of genes within the group was applied: a *P*-value <0.0167 (0.05/3) significance threshold for SKAT-C tests of allopurinol-to-oxypurinol genes. To maximize power all 563 participants meeting inclusion criteria were included in primary analysis. A secondary analysis was carried out on a subset of participants (*n* = 286) with adherence to allopurinol confirmed biochemically (plasma oxypurinol >20 μmol/l). Statistical adjustment for pre-ULT SU was performed in a supplementary analysis because these data were missing in 158/563 (28.1%) participants. To assess effect size directionality, separate multivariable logistic regression models were carried out for each low-frequency or common variant (MAF > 0.1) within *MOCOS* as an independent variable and inadequate response to allopurinol as the dependent variable, adjusting for independent covariables listed above.

## Results

Of 563 participants, 298 (52.9%) had an inadequate response to allopurinol (Table 1). Most participants (92.5%) were male (*n* = 521). Age ranged from 21 to 81. Additionally, 77.3% were of European ancestry (*n* = 435), 10.8% (*n* = 61) of African ancestry and 67 (11.9%) of other ancestries [East Polynesian (*n* = 11), West Polynesian (*n* = 5), East Asian (*n* = 8), South Asian (*n* = 3), Oceanian (*n* = 2) and unknown ancestry (*n* = 37) (Supplementary Table S5, available at *Rheumatology* online)].

**Table 1.** Demographic and clinical data of *n* = 563 participants across separate allopurinol response groups

	Good response	Inadequate response	*P*-value^a^^,^^b^^,^^c^
	(*n* = 265)	(*n* = 298)	
Male sex^a^, *n* (%)	236 (89.1)	285 (95.6)	0.005
Age^b^, mean (s.d.), years	54.2 (11.2)	48.7 (11.2)	<0.001
Ancestry^a^, *n* (%)			
European	214 (80.8)	221 (74.2)	0.011
African	31 (11.7)	30 (10.1)	
Other	20 (7.5)	47 (15.8)	
BMI^b^, mean (s.d.), kg/m^2^	33.0 (7.38)	36.5 (7.56)	<0.001
Tophi^a^, *n* (%)	40 (15.1)	60 (20.1)	0.147
Disease duration^c^, median (Q1, Q3), years	6.8 (2.4, 12.5)	8.2 (3.8, 14.7)	0.028
Number of gout flares^c^ (in previous 12 months), median (Q1, Q3)	4.0 (3.0, 6.0)	4.0 (3.0, 8.0)	0.001
eGFR^c^, median (Q1, Q3), ml/min/1.73 m^2^	84.7 (71.1, 96.6)	88.9 (73.5, 103.1)	0.011
Diuretic use^a^, *n* (%)	46 (17.4)	57 (19.1)	0.665
Pre-ULT Serum urate^c^^,^^d^, median (Q1, Q3), mmol/l	0.523 (0.494, 0.565)	0.577 (0.535, 0.614)	<0.001
Hypertension^a^, *n* (%)	133 (50.2)	164 (55.0)	0.287
Diabetes^a^, *n* (%)	32 (12.1)	37 (12.4)	1
High cholesterol^a^, *n* (%)	101 (38.1)	98 (32.9)	0.228
High triglycerides^a^, *n* (%)	53 (20.0)	44 (14.8)	0.126
History of Stroke^a^, *n* (%)	4 (1.5)	2 (0.7)	0.578
Angina^a^, *n* (%)	9 (3.4)	9 (3.0)	0.989
Myocardial infarction^a^, *n* (%)	8 (3.0)	6 (2.0)	0.622

a Results of Pearson's χ^2^ test shown for categorical variables.

b Mean (s.d.) and results of the two-sample *t*-test parametric test shown for continuous variables with approximately normally distributed data.

c Median (Q1, Q3) and results of Wilcoxon rank-sum non-parametric test shown for continuous variables with non-normal distribution.

d Data for pre-ULT serum urate was missing for 56 (21.1%) controls and 102 (34.2%) gout cases. eGFR: estimated glomerular filtration rate; ULT: urate lowering therapy.

The proportion of men was highest in the inadequate compared with good response group [285 (95.6%) *vs* 236 (89.1%), *P* = 0.005, Table 1]. There was a higher proportion of people of other ancestry (non-European, non-African or unknown ancestry) [47 (15.8%) *vs* 20 (7.5%)], and a lower proportion of people with European ancestry [221 (74.2%) *vs* 214 (80.8%)] or African ancestry [30 (10.1%) *vs* 31 (11.7%)] in the inadequate compared with good response group (*P* = 0.011, Table 1). Mean (s.d.) age was higher in good [54.2 (11.2)] compared with inadequate [48.7 (11.2), *P* < 0.001] responders, as was BMI [mean (s.d.) = 36.5 (7.56) *vs* 33.0 (7.38), *P* < 0.001] and eGFR [median (Q1, Q3) = 88.9 (73.5, 103.1) ml/min/1.73 m^2^  *vs* 84.7 (71.1, 96.6) ml/min/1.73 m^2^, *P* = 0.011]. Tophi were more prevalent in inadequate compared with good responders [60 (20.1%) *vs* 40 (15.1%)], but the difference was non-significant (*P* = 0.15). Poor responders had more flares in the past year compared with good responders [mean (s.d.) = 7.83 (18.9) *vs* 5.09 (4.14), *P* = 0.001]; a longer duration of gout [median (Q1, Q3): 8.2 (3.8, 14.7) years *vs* 6.8 (2.4, 12.5) years, *P* = 0.028]; and higher pre-ULT SU [median (Q1, Q3): 0.577 (0.535, 0.614) mmol/l *vs* 0.523 (0.494, 0.565) mmol/l, *P* < 0.001]. Hypertension [297 (52.8%) participants], diabetes [69 (12.3%) participants], high cholesterol [199 (35.3%)] and diuretic use [103 (18.3%)] were present at similar rates across response groups.

### Rare and common variant combination SKAT-C tests (primary analysis)

The SKAT-C test found an association of common and rare variants within the allopurinol-to-oxypurinol metabolic gene group (*P*_SKAT-C_ = 0.019), but not in the reuptake (*P*_SKAT-C_ = 0.27) or secretory (*P*_SKAT-C_ = 0.78) gene groups, with allopurinol response (Table 2). Based on the association of the allopurinol-to-oxypurinol metabolic gene group with allopurinol response, we tested individual genes within this group (*AOX*, *MOCOS* and *XDH*). There was an association of combined common and rare variants within *MOCOS* (*P*_SKAT-C_ = 0.011), but not within other allopurinol metabolic genes, *AOX1* (*P*_SKAT-C_ = 0.45) or *XDH* (*P*_SKAT-C_ = 0.23), with allopurinol response. The associations of the allopurinol-to-oxypurinol gene group and *MOCOS* with allopurinol response were independent of age, sex, BMI, eGFR, number of gout flares, diuretic use, rs2231142 and the first five genome-wide PC eigenvectors. They were also independent of pre-ULT SU measurements (allopurinol-to-oxypurinol: *P*_SKAT-C_ = 0.038 and *MOCOS*: *P*_SKAT-C_ = 0.032, Supplementary Table S6, available at *Rheumatology* online). The rs2231142 variant associated with inadequate response to allopurinol [odds ratio (OR) (95% CI) = 2.30 (1.62, 3.24), *P* < 0.001], as previously reported in the LASSO dataset (data not shown) [16].

**Table 2.** Results of rare and common variant combination (SKAT_CommonRare or SKAT-C) test for association of with allopurinol response

	Primary analysis (*n* = 563)^a^^,^^c^							Secondary analysis (*n* = 286)^b^^,^^c^						
	Rare^d^	Common^e^^,^^f^	*P*-value^g^^,^^h^	Gene	Rare^e^	Common^e^^,^^f^	*P*-value^g^^,^^i^	Rare^d^	Common^e^^,^^f^	*P*-value^g^^,^^h^	Gene	Rare^d^	Common^e^^,^^f^	*P*-value^g^^,^^i^
Oxypurinol	53	8	**0.019**	*XDH*	20	2	0.227	36	6	**0.002**	*XDH*	13	1	0.574
				*AOX1*	18	1	0.448				*AOX1*	11	1	0.478
				*MOCOS*	15	5	**0.011**				*MOCOS*	12	4	**<0.001**
Reuptake	36	4	0.269	*SLC22A11*	16	0	0.110	22	4	0.075	*SLC22A11*	10	0	0.138
				*SLC2A9*	20	4	0.382				*SLC2A9*	12	4	0.123
Secretory	92	5	0.777	*ABCC4*	25	2	0.395	52	3	0.772	*ABCC4*	13	1	0.516
				*ABCC5*	12	0	0.425				*ABCC5*	5	0	1.000
				*ABCG2*	21	1	0.448				*ABCG2*	13	0	0.798
				*SLC17A1*	8	0	0.738				*SLC17A1*	7	0	0.212
				*SLC17A3*	9	2	0.986				*SLC17A3*	3	2	0.823
				*SLC22A6*	6	0	0.778				*SLC22A6*	2	0	0.388
				*SLC22A8*	11	0	0.303				*SLC22A8*	9	0	0.729

a Primary analysis was carried out on *n* = 563 participant samples (*n* = 290 poor responders and *n* = 261 good responders).

b Secondary analysis was carried out for a subset (286 of 563) of samples (*n* = 148 good and inadequate responders *n* = 138) that had adherence to allopurinol confirmed with plasma oxypurinol measurements.

c SKAT-C test was carried out using default settings.

d Number of rare variants. Rare variant weight [default = β(1, 25)].

e Number of common variants. Common variant weight [default = β(0.5, 0.5)] slowly decreases with increasing MAF.

f Default common variant cut-off was MAF > 1/√2*n* = 0.0301 in primary analysis and 0.042 in secondary analysis.

g Age, sex, BMI, eGFR, diuretic use, number of gout flares in the past year, rs2231142 and the first five principal components were incorporated as covariates in Skat-CommonRare tests.

h Statistical significance, indicated in bold, was defined as *P* < 0.05 for three gene-type (oxypurinol, reuptake and secretory) a priori comparisons.

i Statistical significance, indicated in bold, was defined as *P* < 0.0167 (Bonferroni corrected for a factor of 3) for three allopurinol-oxypurinol conversion genes. *P*-values for reuptake and secretory are purely 'nominal' values not to be interpreted as hypothesis testing, as the gene types were not significant. eGFR: estimated glomerular filtration rate; MAF: minor allele frequency.

### Rare and common variant combination SKAT-C tests in participants with biochemically confirmed adherence to allopurinol (secondary analysis)

Of 303 participants with available plasma samples, 286 had plasma oxypurinol >20 μmol/l, indicating adherence to allopurinol (Supplementary Table S7, available at *Rheumatology* online). Within the subset with oxypurinol-confirmed adherence, the associations of combined rare and common variants in the allopurinol-to-oxypurinol metabolic gene group (*P*_SKAT-C_ = 0.002) and in *MOCOS* (*P*_SKAT-C_ < 0.001) with allopurinol response reached higher levels of statistical significance compared with primary analysis (Table 2). Associations were independent of age, sex, BMI, eGFR, number of gout flares, rs2231142 and the first five genome-wide PC eigenvectors (Table 2), and of pre-ULT SU levels (*P*_SKAT-C_ < 0.001, Supplementary Table S6, available at *Rheumatology* online). *AOX1* (*P*_SKAT-C_ = 0.48) and *XDH* (*P*_SKAT-C_ = 0.57) were not associated with allopurinol response in secondary analysis (Table 2).

### Characterizing individual variants within *MOCOS*

There were 20 variants identified in *MOCOS* in the overall study population (*n* = 563); of these five had MAF above the SKAT default common-rare cut-off ($\frac{1}{\sqrt{2\times \mathrm{sample}\;\mathrm{size}}}$ > 0.030, Table 2). Odds ratio effect sizes of individual low frequency and common variants (MAF > 0.010) within *MOCOS* on allopurinol response were in varying directions (Supplementary Fig. S2A and B, available at *Rheumatology* online). Ten out of 20 *MOCOS* variants in the SKAT-C test were in the PF00266: aminotransferase class V domain (accounting for 153 of the total 644 *MOCOS* minor allele count) (Supplementary Table S8, available at *Rheumatology* online). Three of the 20 *MOCOS* variants were in the PF03473: MOSC C-terminal domain accounting for six of the 644 minor allele count. The remaining variants were proximal to these domains.

## Discussion

Our results suggest that a combination of common and rare variants in *MOCOS* associate with allopurinol response. *MOCOS* encodes molybdenum cofactor sulfurase that sulfurates MOCO cofactor, which is essential for XO/XDH and AOX1 oxidative activity (Fig. 1B) [9]. The association was observed when common and rare variant effects were combined with the widely used SKAT-Common-rare variance-component test [17], which assigns weighted scores based on minor allele frequency and combines variant effects of opposing directions. The signal of association of *MOCOS* common and rare variants with allopurinol response was stronger in a subgroup of participants with adherence to allopurinol determined biochemically by plasma oxypurinol, suggesting that assessment of adherence to treatment improves signal detection. Higher pre-ULT SU and number of gout flares in the past year were also associated with inadequate response to allopurinol, reflecting evidence from the STOP gout trial that baseline SU and characteristics of more severe gout predict ULT failure [14]. Helget and colleagues [14] analysed data of 764 participants with gout from the STOP Gout trial who had been randomized 1:1 to receive allopurinol or febuxostat in a treat-to-target approach. Inclusion criteria were SU ≥0.404 mmol/l at screening and previous allopurinol doses of ≤300 mg. One-third of participants had stage 3 kidney disease, while patients with more advanced kidney disease were excluded. ULT was titrated over 24–48 weeks to reach target SU (< 0.357 mmol/l, or <0.297 mmol/l for those with tophi). Adherence was determined using a patient-recorded medication diary. Helget and colleagues, also found evidence that presence of tophi and diuretic use predict failure to reach target SU on ULT [14], which was not replicated in our data. However, Helget and colleagues used a lower SU target for people with tophi, while we used one SU threshold, irrespective of tophi presence. Inadequate responders were younger, had higher eGFR and were more likely to have non-European/non-African ancestry than good responders in the LASSO cohort, again reflecting the findings of Helget and colleagues [14]. As we previously reported, BMI and male sex strongly associate with inadequate allopurinol response in the LASSO cohort [16]. BMI categories were not associated with ULT response in the STOP gout cohort, and an effect of sex could not be determined [14]. These potential non-genetic predictors of response to allopurinol, particularly those with replicated evidence, may hold clinical value in guiding treatment of gout.

There is replicated evidence that *ABCG2* (rs2231142, Q141K), a missense variant that impacts efflux of urate, predicts inadequate response to allopurinol [15, 16]. The association of *MOCOS* with allopurinol response observed in this study was independent of non-genetic predictors of response and of rs2231142, suggesting *MOCOS* as an additional genetic determinant of allopurinol response, although replication in independent datasets is required to validate our finding. There may be potential to incorporate genetic markers associated with allopurinol response, along with other predictors, in a risk score to inform clinical management of gout. Allopurinol dose prediction tools have been developed that predict the required dose to achieve target SU [31, 32]. These tools include body weight, baseline SU, ethnicity and renal function, and have the possibility of guiding more efficient dose-escalation strategies. Performance of these tools improved with inclusion of *ABCG2* genotype [32]. Whether addition of a *MOCOS* variant genotype score improves performance of allopurinol dose prediction tools remains a question for future research.

It is possible that variation in *MOCOS* affects MOCOS sulfuration activity, thereby indirectly affecting the conversion of allopurinol to oxypurinol by XO and AOX1. Three of the twenty variants detected in *MOCOS* were located in the region encoding the C-terminal domain predicted to be a sulfur-carrier domain that receives and delivers sulfur [33]. Ten *MOCOS* variants were located in the region encoding the aminotransferase class V domain.

Allopurinol-to-oxypurinol metabolic genes also play a crucial role in the thiopurine catabolic pathway. A clinical case study of thiopurine drug-induced toxicity provided convincing genetic, biochemical and enzymatic evidence for a role of *MOCOS* [34].

There are limitations to this study. Not all participants had undergone treat-to-target approach to urate-lowering by ULT. We did not have access to a suitable replication cohort to determine reproducibility of the results.

In conclusion, an association was observed for rare and common variants in the allopurinol-to-oxypurinol conversion gene, *MOCOS*. The statistical strength of the signal was improved with more accurate phenotyping of adherence to allopurinol therapy. These results support the idea that additional genetic factors play a part in determining response to allopurinol and point to a mechanism involving allopurinol-to-oxypurinol conversion.

## Supplementary Material

keae420_Supplementary_Data

## Data Availability

The individual-level data cannot be made publicly available due to consent restrictions, but data may be available from the corresponding author on reasonable request.
