# Supplementary material for: Association of rare and common genetic variants in MOCOS with inadequate response to allopurinol
Source: Rheumatology (Oxford). 2024 Aug 13;63(11):3025–32. doi: 10.1093/rheumatology/keae420 (PMC11534095; doi:10.1093/rheumatology/keae420)
Supplement: keae420_Supplementary_Data [file keae420_supplementary_data.docx]

# **Supplementary Materials**

**Supplementary Table S1. Inclusion and exclusion criteria in LASSO trial as shown in Table 1 of Becker et al. 2015 (1)**

| **Inclusion criteria** |
| --- |
| 1. 18-85 years of age |
| 2. Male or female; females of childbearing potential who were sexually active must have agreed to use adequate contraception (as determined by the Investigator) and could neither be pregnant nor lactating from screening throughout the duration of the study |
| 3. Patient met the diagnosis of gout according to the American Rheumatism Association (ARA) Criteria for the Classification of Acute Arthritis of Primary Gout |
| 4. Patients not on a urate lowering therapy ULT must have had a serum urate (SU) ≥ 0.476 mmol/L at screening |
| 5. Patients on concomitant ULT must have had SU ≥ 387 mmol/L at screening |
| 6. Patient must have had at least 2 gout flares in the past year |
| 7. Patient was willing and able to give informed consent and adhere to visit/protocol schedules |
| **Exclusion criteria** |
| 1. Baseline SU < 0.387 mmol/L |
| 2. More than 14 drinks of alcohol per week [e.g., 1 drink = 5 oz (150mL) of wine, 12oz (360mL) of beer, or 1.5oz (45mL) of spirits] |
| 3. History or suspicion of drug abuse |
| 4. History of myositis or rhabdomyolysis |
| 5. History of autoimmune disease requiring systemic treatment |
| 6. Known or suspected human immunodeficiency virus (HIV), hepatitis C antibody (HCV), or hepatitis B antibody (HBsAg) infection |
| 7.History of malignancy within the previous 5 years (with the exception of non-melanoma skin cancer that has been treated with no evidence of recurrence, treated cervical dysplasia, or treated in situ Grade 1 cervical cancer) |
| 8. Myocardial infarction, unstable angina, New York Heart Association (NYHA) class III or IV heart failure, or stroke within the last 12 months |
| 9. Uncontrolled hypertension (systolic pressure above 160mmHg or diastolic pressure above 95mm Hg) |
| 10. Estimated creatinine clearance at screening < 30 mL/min by Cockcroft Gault formula |
| 11. Kidney or other organ transplant |
| 12. Haemoglobin < 10g/dL (males) or < 9 g/dL (females) |
| 13. Alanine amino transferase (ALT) or aspartate amino transferase (AST) > 2 upper limit of normal (ULN) |
| 14. Gamma-glutamyl transferase (GGT) > 3 upper limit of normal (ULN) |
| 15.Active pepticulcer disease requiring treatment |
| 16.History of xanthinuria, active liver disease, or hepatic dysfunction |
| 17.Requires therapy with any others serum urate-lowering medication, other than allopurinol |
| 18. Unable to take gout flare prophylaxis of either colchicine or NSAID due to contraindication (e.g., toxicity, renal function, and use of contra-indicated medications) |
| 19. Received an investigational medication within 8 weeks or 5 half-lives (whichever is longer) prior to the screening visit for this study |
| 20. Previously participated in a clinical study involving lesinurad (RDEA594) or RDEA806 |
| 21.Previously received pegloticase |
| 22. Known hypersensitivity or allergy to allopurinol |
| 23. Any other medical or psychological condition that, in the opinion of the Investigator and/or Medical Monitor, might create undue risk to the patient or interfere with the patient's ability to comply with the protocol requirements, or to complete the study |

ARA, American Rheumatism Association; HIV, human immunodeficiency virus; HCV, hepatitis C antibody; HBsAg, hepatitis B antibody; GGT, Gamma-glutamyl transferase; NYHA , New York Heart Association; SU, serum urate; ULT, urate lowering therapy

Supplementary Table S2. Demographic and clinical data of n=667 sequenced subset and the overall LASSO trial

|  | Not sequenced (N=1077) | Whole Genome Sequencing (N=667) | Overall (N=1744) |
| --- | --- | --- | --- |
| **Sex** |  |  |  |
| Female | 67 (6.2%) | 51 (7.6%) | 118 (6.8%) |
| Male | 1003 (93.1%) | 614 (92.1%) | 1617 (92.7%) |
| Missing | 7 (0.7%) | 2 (0.3%) | 9 (0.5%) |
| **Age** |  |  |  |
| Mean (SD) | 51.2 (12.0) | 51.7 (11.7) | 51.4 (11.9) |
| Median [Min, Max] | 51.0 [21.0, 84.0] | 51.0 [21.0, 84.0] | 51.0 [21.0, 84.0] |
| Missing | 7 (0.7%) | 2 (0.3%) | 9 (0.5%) |
| **Race** |  |  |  |
| American Indian or Alaska Native | 12 (1.1%) | 0 (0%) | 12 (0.7%) |
| Asian | 62 (5.8%) | 33 (4.9%) | 95 (5.4%) |
| Black or African American | 96 (8.9%) | 71 (10.6%) | 167 (9.6%) |
| Native Hawaiian or Other Pacific Islander | 38 (3.5%) | 17 (2.5%) | 55 (3.2%) |
| Other | 89 (8.3%) | 21 (3.1%) | 110 (6.3%) |
| White | 773 (71.8%) | 523 (78.4%) | 1296 (74.3%) |
| Missing | 7 (0.7%) | 2 (0.3%) | 9 (0.5%) |
| **BMI (kg/m2)** |  |  |  |
| Mean (SD) | 34.2 (7.48) | 34.7 (7.68) | 34.4 (7.56) |
| Median [Min, Max] | 32.7 [16.2, 91.7] | 33.2 [14.2, 86.0] | 32.9 [14.2, 91.7] |
| Missing | 13 (1.2%) | 4 (0.6%) | 17 (1.0%) |
| **Duration of gout diagnosis (years)** |  |  |  |
| Mean (SD) | 10.4 (9.31) | 10.1 (9.08) | 10.3 (9.22) |
| Median [Min, Max] | 7.50 [0, 68.2] | 7.80 [0, 46.8] | 7.70 [0, 68.2] |
| Missing | 1 (0.1%) | 0 (0%) | 1 (0.1%) |
| **Number of flares in past year** |  |  |  |
| 2-4 | 447 (41.5%) | 260 (39.0%) | 707 (40.5%) |
| >=5 | 455 (42.2%) | 289 (43.3%) | 744 (42.7%) |
| Missing | 175 (16.2%) | 118 (17.7%) | 293 (16.8%) |

**
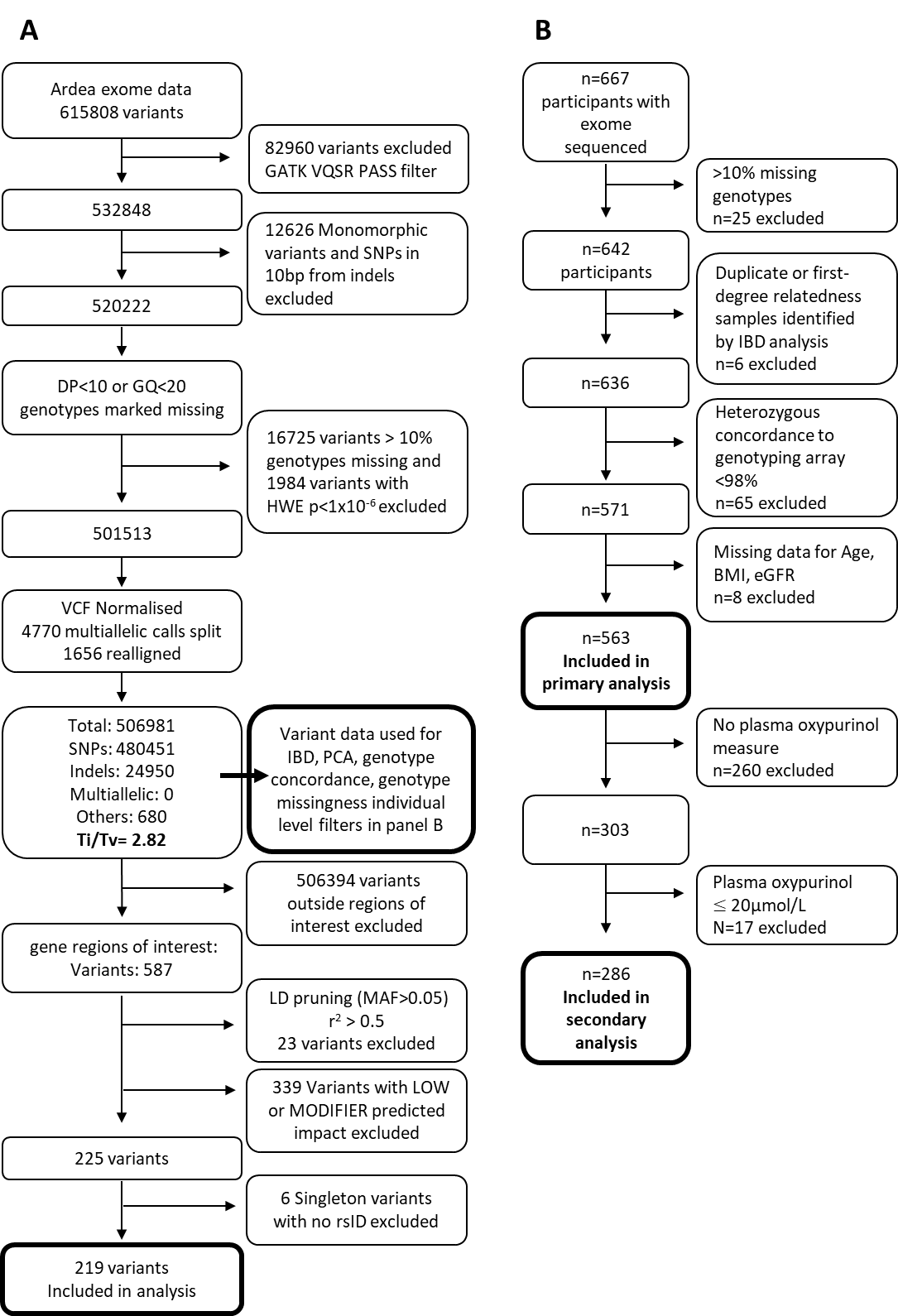
Supplementary Figure S1. Flowchart of variant level filtering steps (A) and individual level filtering steps (B) that were applied as quality control** to sequencing data from n=667 samples. Variant call format files were filtered to contain exonic variants in regions of the truseq exome target prior to filtering steps shown. IBD, principal component analysis: PCA, genotype concordance analysis, percentage of missing genotypes per sample.

**Supplementary Table S3. Genomic coordinates of genes included in analysis**

| gene | Chromosome | Position a | Position b | Gene Function |
| --- | --- | --- | --- | --- |
| *ABCC4* | 13 | 95672083 | 95953687 | secretory |
| *ABCC5* | 3 | 183637722 | 183735803 | secretory |
| *ABCG2* | 4 | 89011416 | 89152474 | secretory |
| *SLC17A1* | 6 | 25783125 | 25832287 | secretory |
| *SLC17A3* | 6 | 25833294 | 25882514 | secretory |
| *SLC22A6* | 11 | 62703857 | 62752455 | secretory |
| *SLC22A8* | 11 | 62756626 | 62783311 | secretory |
| *SLC22A12* | 11 | 64358113 | 64369820 | reuptake |
| *SLC2A9* | 4 | 9772777 | 10056560 | reuptake |
| *SLC22A11* | 11 | 64323098 | 64340347 | reuptake |
| *AOX1* | 2 | 201450591 | 201541787 | Allo-to-oxy |
| *MOCOS* | 18 | 33767482 | 33852120 | Allo-to-oxy |
| *XDH* | 2 | 31557187 | 31637581 | Allo-to-oxy |

Gene regions are between position a and position b. Allo-to-oxy = allopurinol to oxypurinol conversion genes; Reuptake = reuptake transporter genes; Secretory= Secretory transporter genes

Supplementary Table S4. Putative impact and annotation (effects) of variants in gene regions of interest

| Impact | Annotation | N markers |
| --- | --- | --- |
| **HIGH** | **frameshift_variant** | **6** |
| **HIGH** | **splice_donor_variant&intron_variant** | **2** |
| **HIGH** | **stop_gained** | **10** |
| **MODERATE** | **conservative_inframe_deletion** | **1** |
| **MODERATE** | **disruptive_inframe_deletion** | **1** |
| **MODERATE** | **missense_variant** | **193** |
| **MODERATE** | **missense_variant&splice_region_variant** | **6** |
| LOW | 5_prime_UTR_premature_start_codon_gain_variant | 1 |
| LOW | splice_region_variant&intron_variant | 18 |
| LOW | splice_region_variant&synonymous_variant | 6 |
| LOW | synonymous_variant | 153 |
| MODIFIER | 3_prime_UTR_variant | 6 |
| MODIFIER | 5_prime_UTR_variant | 9 |
| MODIFIER | downstream_gene_variant | 1 |
| MODIFIER | intergenic_region | 2 |
| MODIFIER | intron_variant | 126 |
| MODIFIER | upstream_gene_variant | 5 |
| Putative impact and annotation are subfields of ANN field from SnpEFF | | |

Supplementary Table S5. Ancestry of n=563 participants across separate allopurinol response groups

|  | Good response (N=265) | Inadequate response (N=298) | Overall (N=563) |
| --- | --- | --- | --- |
| **Ancestry** |  |  |  |
| African | 31 (11.7%) | 30 (10.1%) | 61 (10.8%) |
| East Asian | 4 (1.5%) | 4 (1.3%) | 8 (1.4%) |
| East Polynesian | 3 (1.1%) | 8 (2.7%) | 11 (2.0%) |
| European | 214 (80.8%) | 221 (74.2%) | 435 (77.3%) |
| Oceanian | 2 (0.8%) | 0 (0%) | 2 (0.4%) |
| South Asian | 2 (0.8%) | 1 (0.3%) | 3 (0.5%) |
| West Polynesian | 0 (0%) | 5 (1.7%) | 5 (0.9%) |
| Unknown | 9 (3.4%) | 29 (9.7%) | 38 (6.7%) |
|  | | | |

**Supplementary Table S6. SKAT-C test with additional statistical adjustment for pre-ULT serum urate**

|  | Primary analysis^a^ | | | | | Secondary Analysis^b^ | | | | |
| --- | --- | --- | --- | --- | --- | --- | --- | --- | --- | --- |
|  | Number of rare variants^c^ | Number of common variants^d,e^ | P-value^f,g^ | Gene type | P-value^f,g^ | Number of rare variants^c^ | Number of common variants^d,e^ | P-value^f,g^ | Gene type | P-value^f,g^ |
| *XDH* | 18 | 1 | 0.345 | Allopurinol to oxypurinol | 0.038 | 13 | 0 | 0.406 | Allopurinol to oxypurinol | **< 0.001** |
| *AOX1* | 15 | 1 | 0.228 |  |  | 8 | 1 | 0.200 |  |  |
| *MOCOS* | 11 | 5 | 0.032 |  |  | 6 | 4 | **< 0.001** |  |  |

^a^Subset with preULT serum urate measure available (n=405/563, 71.9%). ^b^Subset with preULT serum urate measure available and adherence determined by plasma oxypurinol (n = 197/563). SKAT-C test was carried out using default settings: ^c^Rare variant weight (default=c(1, 25)); ^d^Common variant cut-off was MAF > 1/√2n (0.035 in n=405 subset and 0.05 in n=197 subset);  ^e^Common variant weight (default=c(0.5,0.5)) slowly decreases with increasing MAF;  ^f^Age, sex, BMI, EGFR, hypertension, number of gout flares, diuretic use, rs2231142, pre-ULT serum urate and the first 5 principal components were incorporated as covariates in Skat-CommonRare tests; ^g^Statistical significance was corrected for multiple testing and defined as p < 1.32 x 10^‑3^ and indicated in bold.

**Supplementary Table S7. Demographic and clinical data of n=286 participants across separate allopurinol response groups**

|  | Good response (N=148) | Inadequate response (N=138) | P-value^a^ |
| --- | --- | --- | --- |
| **Age** |  |  |  |
| Mean (SD) | 53.5 (11.3) | 50.3 (11.0) | 0.032 |
| Median [Min, Max] | 53.0 [28.0, 80.0] | 50.0 [24.0, 79.0] |  |
| **Sex** |  |  |  |
| Female | 8 (5.4%) | 5 (3.6%) | 0.661 |
| Male | 140 (94.6%) | 133 (96.4%) |  |
| **Ancestry** |  |  |  |
| European | 121 (81.8%) | 107 (77.5%) | 0.047 |
| African | 15 (10.1%) | 8 (5.8%) |  |
| Other | 12 (8.1%) | 23 (16.7%) |  |
| **BMI (kg/m2)** |  |  |  |
| Mean (SD) | 33.4 (6.24) | 37.8 (7.57) | <0.001 |
| Median [Min, Max] | 32.3 [14.2, 60.1] | 37.1 [23.7, 62.2] |  |
| **Tophi** |  |  |  |
| No | 131 (88.5%) | 117 (84.8%) | 0.451 |
| Yes | 17 (11.5%) | 21 (15.2%) |  |
| **Disease duration** |  |  |  |
| Mean (SD) | 9.88 (9.46) | 10.8 (9.29) | 0.16 |
| Median [Min, Max] | 6.95 [0, 42.9] | 8.05 [0, 46.8] |  |
| **Number of flares** |  |  |  |
| Mean (SD) | 5.31 (4.57) | 5.58 (4.24) | 0.153 |
| Median [Min, Max] | 4.00 [2.00, 30.0] | 4.00 [2.00, 24.0] |  |
| **eGFR (mL/min/1.73 m2)** |  |  |  |
| Mean (SD) | 83.0 (18.6) | 85.1 (20.8) | 0.264 |
| Median [Min, Max] | 83.8 [37.5, 133] | 87.1 [5.23, 129] |  |
| **Diuretic use** |  |  |  |
| No | 121 (81.8%) | 106 (76.8%) | 0.375 |
| Yes | 27 (18.2%) | 32 (23.2%) |  |
| **Pre-ULT Serum urate (mmol/L)** |  |  |  |
| Mean (SD) | 0.545 (0.0627) | 0.566 (0.0627) | 0.004 |
| Median [Min, Max] | 0.529 [0.446, 0.803] | 0.559 [0.422, 0.738] |  |
| Missing | 32 (21.6%) | 57 (41.3%) |  |
| **Hypertension** |  |  |  |
| No | 77 (52.0%) | 54 (39.1%) | 0.039 |
| Yes | 71 (48.0%) | 84 (60.9%) |  |
| **Diabetes** |  |  |  |
| No | 135 (91.2%) | 117 (84.8%) | 0.134 |
| Yes | 13 (8.8%) | 21 (15.2%) |  |
| **High Cholesterol** |  |  |  |
| No | 88 (59.5%) | 94 (68.1%) | 0.162 |
| Yes | 60 (40.5%) | 44 (31.9%) |  |
| **High triglycerides** |  |  |  |
| No | 117 (79.1%) | 116 (84.1%) | 0.349 |
| Yes | 31 (20.9%) | 22 (15.9%) |  |
| **History of Stroke** |  |  |  |
| No | 144 (97.3%) | 138 (100%) | 0.15 |
| Yes | 4 (2.7%) | 0 (0%) |  |
| **Angina** |  |  |  |
| No | 142 (95.9%) | 133 (96.4%) | 1 |
| Yes | 6 (4.1%) | 5 (3.6%) |  |
| **Myocardial infarction** |  |  |  |
| No | 142 (95.9%) | 137 (99.3%) | 0.15 |
| Yes | 6 (4.1%) | 1 (0.7%) |  |
| ^a^Pearson's 𝛘^2^ test for categorical variables and Wilcoxon rank sum test for continuous variables | | | |


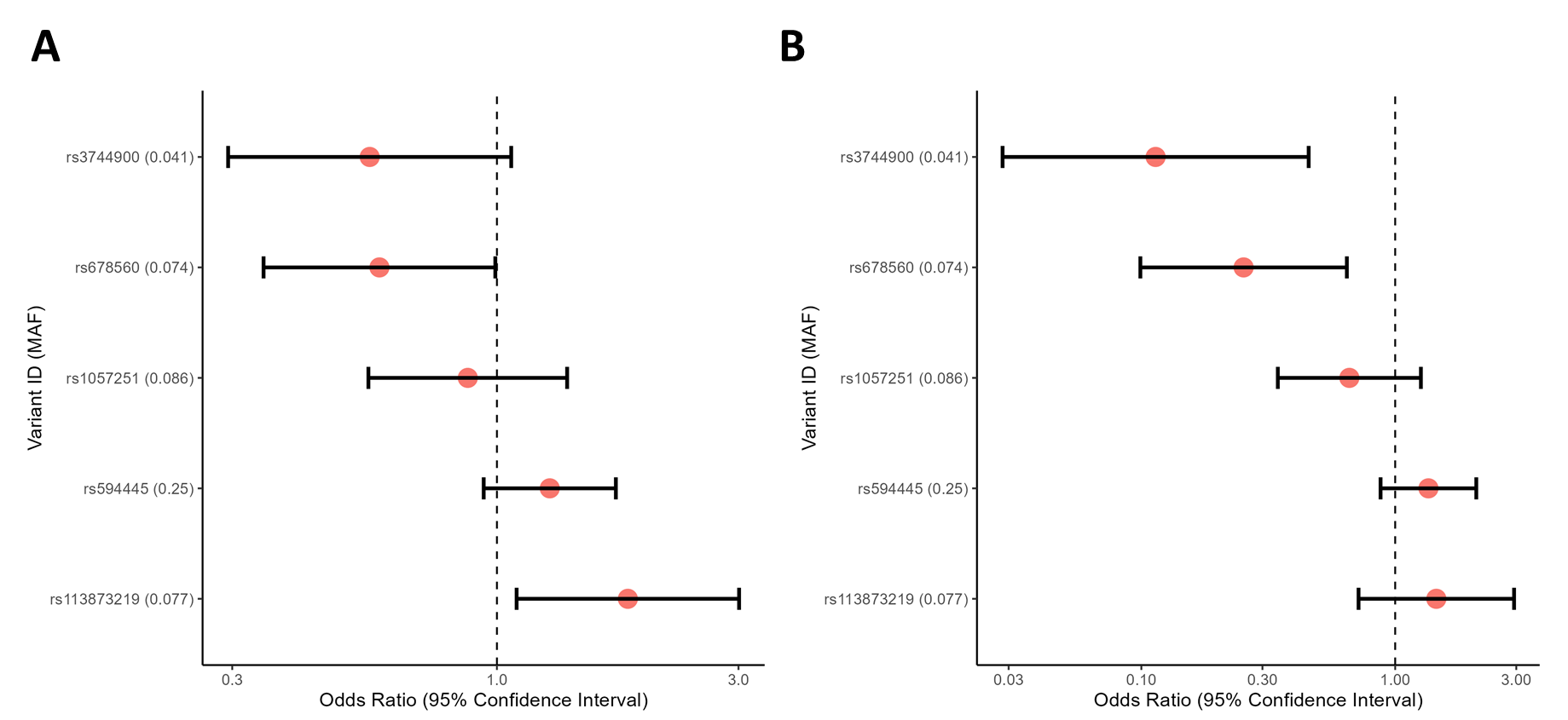


**Supplementary Figure S2. Forest plot of logistic regression estimates (x axis, Odds ratios and nominal 95% confidence intervals without adjustment for multiple comparisons) for association with inadequate response to allopurinol of common variants (MAF > 0.01) in *MOCOS* in overall study cohort (A) and in subset of participants with adherence to allopurinol confirmed by plasma oxypurinol (B). Variant rsIDs and minor allele frequencies (shown in brackets) on the Y axis. MAF: minor allele frequency.**

Supplementary Table S8. Variants in MOCOS

| rsID | CHROM | POS | REF | ALT | MAC^a^ | MAF | HGVS.p | Pfam Domain | Interactions | CADD score |
| --- | --- | --- | --- | --- | --- | --- | --- | --- | --- | --- |
| rs576409195 | 18 | 33,767,509 | G | C | 2.0585 | 0.0016 | p.Gly3Arg | - | - | 16.39 |
| rs113873219 | 18 | 33,767,568 | C | A | 88.906 | 0.0772 | p.Ser22Arg | - | - | 14.18 |
| rs75201776 | 18 | 33,779,683 | A | G | 1 | 0.0023 | p.Thr113Ala | PF00266: Aminotransferase class V | - | 21.5 |
| rs3744900 | 18 | 33,779,705 | G | A | 48 | 0.0413 | p.Ser120Asn | PF00266: Aminotransferase class V | is involved in contacts to ligand | 23.3 |
| rs150556770 | 18 | 33,779,858 | C | T | 1 | 0.0008 | p.Pro171Leu | PF00266: Aminotransferase class V | Involved in contacts to protein | 21.5 |
| rs540967 | 18 | 33,779,896 | A | G | 7 | 0.0068 | p.Ser184Gly | PF00266: Aminotransferase class V | - | 1.205 |
| rs623558 | 18 | 33,780,020 | A | G | 7 | 0.0068 | p.His225Arg | PF00266: Aminotransferase class V | - | 5.992 |
| rs150272391 | 18 | 33,780,188 | C | T | 2 | 0.0015 | p.Ala281Val | PF00266: Aminotransferase class V | - | 0.035 |
| rs139002197 | 18 | 33,780,236 | C | T | 2 | 0.0015 | p.Ala297Val | PF00266: Aminotransferase class V | Involved in contacts to protein | 12.42 |
| rs678560 | 18 | 33,785,093 | G | A | 81 | 0.0742 | p.Val358Met | PF00266: Aminotransferase class V | - | 0.041 |
| rs201070779 | 18 | 33,795,494 | G | A | 1 | 0.0015 | p.Gly451Arg | PF00266: Aminotransferase class V | - | 28.8 |
| rs35980997 | 18 | 33,795,608 | C | T | 3 | 0.0037 | p.Arg489Cys | PF00266: Aminotransferase class V | - | 9.241 |
| rs145294379 | 18 | 33,795,732 | C | T | 2 | 0.0022 | p.Ser530Leu | - | - | 4.073 |
| rs114735250 | 18 | 33,795,867 | T | C | 4 | 0.0052 | p.Leu575Pro | - | - | 4.467 |
| rs594445 | 18 | 33,831,189 | C | A | 280 | 0.2496 | p.His703Asn | - | - | 20.9 |
| rs77772031 | 18 | 33,840,005 | A | G | 4.0071 | 0.0045 | p.Glu759Gly | PF03473: MOSC domain | - | 24.1 |
| rs776357546 | 18 | 33,840,056 | G | A | 1 | 0.0007 | p.Arg776His | PF03473: MOSC domain | - | 31 |
| rs768710712 | 18 | 33,840,131 | G | A | 1 | 0.0007 | p.Arg801His | PF03473: MOSC domain | - | 0.036 |
| rs79823152 | 18 | 33,848,580 | G | A | 5 | 0.0045 | p.Val867Met | - | - | 18.39 |
| rs1057251 | 18 | 33,848,581 | T | C | 103 | 0.0862 | p.Val867Ala | - | - | 23.4 |

CHROM: chromosome, POS: position; REF: reference allele; ALT: alternate allele; ^a^MAC: Minor allele count across samples in SKAT-C test, missing genotypes were imputed based on Hardy-Weinberg equilibrium; MAF: Minor allele frequency; HGVS.p: Amino acid change; CADD score: Phred-scaled CADD score predicted measure of deleteriousness; All variants were missense variants

References

1. Becker MA, Fitz-Patrick D, Choi HK, et al. An open-label, 6-month study of allopurinol safety in gout: The LASSO study. Semin Arthritis Rheum 2015;45:174-83.
